# Differences between physical therapist ratings, self-ratings, and posturographic measures when assessing static balance exercise intensity

**DOI:** 10.3389/fresc.2023.1096171

**Published:** 2023-05-11

**Authors:** Jamie Ferris, Jonathan Zwier, Wendy J. Carender, Kathleen H. Sienko

**Affiliations:** ^1^ Department of Mechanical Engineering, University of Michigan, Ann Arbor, MI, United States; ^2^ Michigan Balance Vestibular Testing and Rehabilitation, Department of Otolaryngology, Michigan Medicine, Ann Arbor, MI, United States

**Keywords:** balance, balance training, intensity, self-assessment, IMU, wearable sensors

## Abstract

**Introduction:**

In order for balance therapy to be successful, the training must occur at the appropriate dosage. However, physical therapist (PT) visual evaluation, the current standard of care for intensity assessment, is not always effective during telerehabilitation. Alternative balance exercise intensity assessment methods have not previously been compared to expert PT evaluations. The aim of this study was therefore to assess the relationship between PT participant ratings of standing balance exercise intensity and balance participant self-ratings or quantitative posturographic measures.

**Methods:**

Ten balance participants with age or vestibular disorder-related balance concerns completed a total of 450 standing balance exercises (three trials each of 150 exercises) while wearing an inertial measurement unit on their lower back. They provided per-trial and per-exercise self-ratings of balance intensity on a scale from 1 (steady) to 5 (loss of balance). Eight PT participants reviewed video recordings and provided a total of 1,935 per-trial and 645 per-exercise balance intensity expert ratings.

**Results:**

PT ratings were of good inter-rater reliability and significantly correlated with exercise difficulty, supporting the use of this intensity scale. Per-trial and per-exercise PT ratings were significantly correlated with both self-ratings (r = 0.77–0.79) and kinematic data (r = 0.35–0.74). However, the self-ratings were significantly lower than the PT ratings (difference of 0.314–0.385). Resulting predictions from self-ratings or kinematic data agreed with PT ratings approximately 43.0–52.4% of the time, and agreement was highest for ratings of a 5.

**Discussion:**

These preliminary findings suggested that self-ratings best indicated two intensity levels (i.e., higher/lower) and sway kinematics were most reliable at intensity extremes.

## 1. Introduction

Balance ability deteriorates with age (1–3), leading to increased fall risk and fall prevalence in healthy older adults (4). Balance can also be impaired by the presence of sensory or neurologic disorders such as peripheral sensory loss (5, 6), vestibular disorder (6–8), multiple sclerosis (9–11), cerebrovascular accident (12), traumatic brain injury (13, 14), and Parkinson's disease (15, 16). Low balance confidence and fear of falling are associated with increased fall risk (17, 18), anxiety (19–21), depression (19, 20), and institutionalization (21, 22), and with decreased activity (3, 19, 23), social participation (23, 24), and quality of life (19, 23).

Balance training, which is traditionally performed in a clinical setting with the instruction of a physical therapist (PT), can improve balance ability when it occurs at the appropriate dosage such that the training is at or near the limits of an individual's ability (25–27). Dosage has been described as the combination of frequency, intensity, time, and type (FITT) where frequency refers to the regularity of training sessions, intensity refers to “the degree of challenge to the balance control system relative to the capacity of the individual to maintain balance” (28), time refers to the duration of each exercise and of the session, and type refers to the exercise itself (29–31). While frequency, time, and type are easily quantified (29, 31), intensity depends on both the difficulty of the exercise and the balancing ability of the individual, reflecting how challenging an exercise is for a particular individual at a specific time; it is therefore more difficult to assess (29–31). Unlike aerobic (e.g., percent of maximum heart rate) or resistance (e.g., percent of the 1-repetition maximum) exercise intensity (32), there is no standard assessment for balance exercise intensity. Most PTs visually observe the patient, employing their expert assessment to qualitatively adjust task intensity (33, 34). However, visual expert assessment is more difficult during increasingly common telerehabilitation or home-based training during which a PT is not physically present.

Telerehabilitation has grown in popularity as a solution to both misdistribution of physical therapy services (35, 36) and public health concerns such as those posed by COVID-19 (37–40). Access to traditional physical therapy services is increasingly limited. It is predicted that by 2030, the United States alone will have a shortage of 140,000 PTs, resulting in the majority of patients having difficulty accessing necessary physical therapy services (41–43). Moreover, PTs are less available in low-income countries (35, 44, 45), where the majority of people with disabilities live (35). Availability of physical therapy is also lower in rural settings (35, 36, 44), where chronic conditions amenable to physical therapy are prevalent (36). In other countries such as Singapore, PTs are concentrated in acute-care facilities, leading to lesser availability in post-acute care sites (44).

While telerehabilitation may improve access to care, only 29% of PTs in a 2022 vestibular rehabilitation study expressed that telerehabilitation was as effective as in-person care (37); 92% of PTs in the same study reported that technology limitations affected their ability to view patients' bodies remotely. PTs have also reported that inappropriate lighting and patient positioning with respect to the camera can negatively affect clinical decision making (37, 46). Relatedly, patients have reported a lack of appropriate technology including computers with cameras (39), making visual assessment impossible in some cases.

In addition to telerehabilitation, home exercise programs may complement existing affordable physical therapy options available to patients, which have been limited in their duration. For example, until recently, public insurance for older adults in the United States (i.e., Medicare) covered only approximately 14–16 outpatient physical therapy visits [44]. In Singapore, only select follow-up rehabilitation services can be paid for with compulsory medical savings. And in Bangladesh, public funding for physical therapy is limited (44). As employment rates are lower for people with disabilities, extended physical therapy care is often financially inaccessible (35, 44). However, training without a professional's guidance has been shown to be less effective than supervised training (47, 48).

In contexts where visual assessment of balance task intensity is difficult such as telerehabilitation and home-based training, PTs may time exercises, employ quantitative posturography, or ask for patient self-assessments (38). Timing standing balance exercises may reflect the intensity of a task, but additional performance aspects such as arm movements or trunk sway may be of importance, and there may be a ceiling effect if task duration is limited (e.g., 30 s) (49).

Quantitative posturography, which is possible with inertial measurement units (IMUs) found in ubiquitous technology such as smart phones, can measure an individual's center of mass during standing balance and may therefore be indicative of task intensity (50–55). Alsubaie et al. compared kinematic measures to adults' self-ratings of intensity using 5 and 10-point scales, finding correlations of >0.6 in healthy adults and older adults (53) and >0.5 in adults with vestibular disorders (56) completing static standing balance exercises (i.e., exercises with the feet in place and a goal of maintaining quiet posture) (57). However, the relationships between the kinematic measures and self-ratings with PT (expert) assessments were not evaluated.

Self-report measures of intensity are analogous to the Borg Rating of Perceived Exertion for aerobic exercise (58) or the OMNI Resistance Exertion Scale for resistance exercise (59, 60). Espy et al. proposed a 10-point self-rating scale modeled after the Rate of Perceived Exertion and found that self-ratings of intensity are independent of heart rate in adults playing Wii games, supporting that the scale measured intensity beyond solely aerobic exertion (31). Shenoy et al. examined the same scale in people post-stroke and reported good test-retest reliability, good-to-excellent correlation with self-ratings of perceived challenge, and good-to-excellent correlation with clinical balance test scores (61). Farlie et al. proposed both 13-element and 5-point self-rating scales (28), reporting strong correlation (r = 0.70) between the scales in older adults completing standing balance tasks. However, they also reported that the 5-point scale was of poor person reliability and unable to statistically detect differences in intensity (62). While these studies suggest that self-assessments hold promise as measures of balance task intensity, it is important to understand the relationship between self-report measures and PT expert assessments as the current standard of care. Farlie et al. reported a strong correlation (r = 0.70) between self-rater and PT 5-point ratings of intensity, but they did not further comment on the differences (62). The relationship between self-rater and PT assessments of intensity is still poorly understood.

While quantitative posturography and self-assessment have both shown promise as methods for remote assessment of balance task intensity, few comparisons have been made to conventional visual assessment by expert PTs. This study therefore aimed to further assess the differences between PT expert assessments of balance intensity and self-assessments and kinematic measures.

## 2. Materials and methods

In this study on balance exercise intensity assessment, PT participant intensity ratings (i.e., PT ratings) were compared to balance participant intensity self-ratings (i.e., self-ratings) and kinematic measures.

### 2.1. Participants

Two types of participants completed this study: balance participants with age or vestibular disorder-related balance concerns and PT participants. Balance participants completed standing balance exercises while wearing an IMU, and they provided self-ratings of balance intensity. PT participants reviewed video recordings of the balance participants and provided ratings of balance intensity. 450 balance exercise trials were completed by 10 adult balance participants. Six older adult balance participants (2 F, 4 M, 69.0 ± 3.6 years) self-reported to be generally healthy with no muscular or neurological disorders, and four balance participants (2 F, 2 M, 61.7 ± 25.6 years) self-reported vestibular dysfunction. In addition, 1,935 intensity ratings were provided by eight PT participants who specialized in the treatment of balance disorders (16 ± 10 years of experience). The number of participants and resulting quantity of data is consistent with a number of related previously published studies (63–66). The study was approved by a University of Michigan Institutional Review Board (HUM00086479), and all participants gave written informed consent in accordance with the Helsinki Declaration.

### 2.2. Intensity rating scales

Both PT and balance participants were provided a 5-point intensity rating scale [see Table 1; also reported by Bao et al., Bao et al., and Kamran et al. (50–52)]. The scale was informed by a scale developed by Espy et al. (31). Different descriptors were provided to PT and balance participants with the intent of capturing similar performance aspects while using familiar terminology.

**Table 1:** The 1–5 balance intensity scale provided to PT and balance participants.

Rating	Descriptions for Balance Participants	Descriptions for PT Participants
1	I feel completely steady	Independent with no sway
2	I feel a little unsteady or off-balance	Supervision with minimal sway
3	I feel somewhat unsteady, or like I may lose my balance	Close supervision with moderate sway
4	I feel very unsteady, or like I definitely will lose my balance	Requires physical assistance or positive stepping strategy after 15 s
5	I lost my balance	Unable to maintain position with assist or step out in the first 15 s of the exercise

### 2.3. Procedures

#### 2.3.1. Completion of balance exercises

Each balance participant completed 15 static standing balance exercises during which the participant was asked to maintain their feet in place and maintain a quiet posture. The exercises were selected from 54 possible options resulting from variations in surface (firm, foam), stance (feet apart, feet together, partial heel to toe, heel to toe, single leg stance), visual input (eyes open, eyes closed), and head movement (none, pitch, yaw) (57). Six exercises were excluded due to excessive difficulty for the study population (e.g., combinations of a single leg stance, eyes closed, and yaw or pitch head movements; combinations of a single leg stance and a foam surface). The exercises were selected so that each participant performed exercises spanning the intensity scale (i.e., from 1 to 5). Three 30-second trials were completed for each exercise resulting in a total of 45 total trials per balance participant. Because the active exercise duration (15 exercises × 3 trials ×  30 s = 22 min 30 s) was less than the optimal duration of a balance training session (31–45 min), fatigue was expected to be minimal (25).

For the duration of testing, the balance participant wore a single six-degree-of-freedom IMU (MTx, XSens Inc, Eschende, Netherlands) on an elastic belt approximately positioned over the L4/L5 vertebrae level dorsal to the spine. IMU data were collected at 100 Hz using custom software, and the trunk sway angles were extracted using XSens' proprietary sensor fusion algorithm. Balance participants also wore an overhead harness positioned to provide no support unless the balance participant experienced a loss of balance, and they were provided a handrail for additional safety. They were instructed to touch the handrail, step out of position, or rely on the harness only when needed, continuing the trial after use of these additional supports. After each trial, the balance participant provided a per-trial self-rating of intensity. Additionally, after each exercise (i.e., collection of three trials), the balance participant provided a per-exercise self-rating of the overall intensity of that exercise.

#### 2.3.2. Evaluation by physical therapist participants

**Figure 1:**
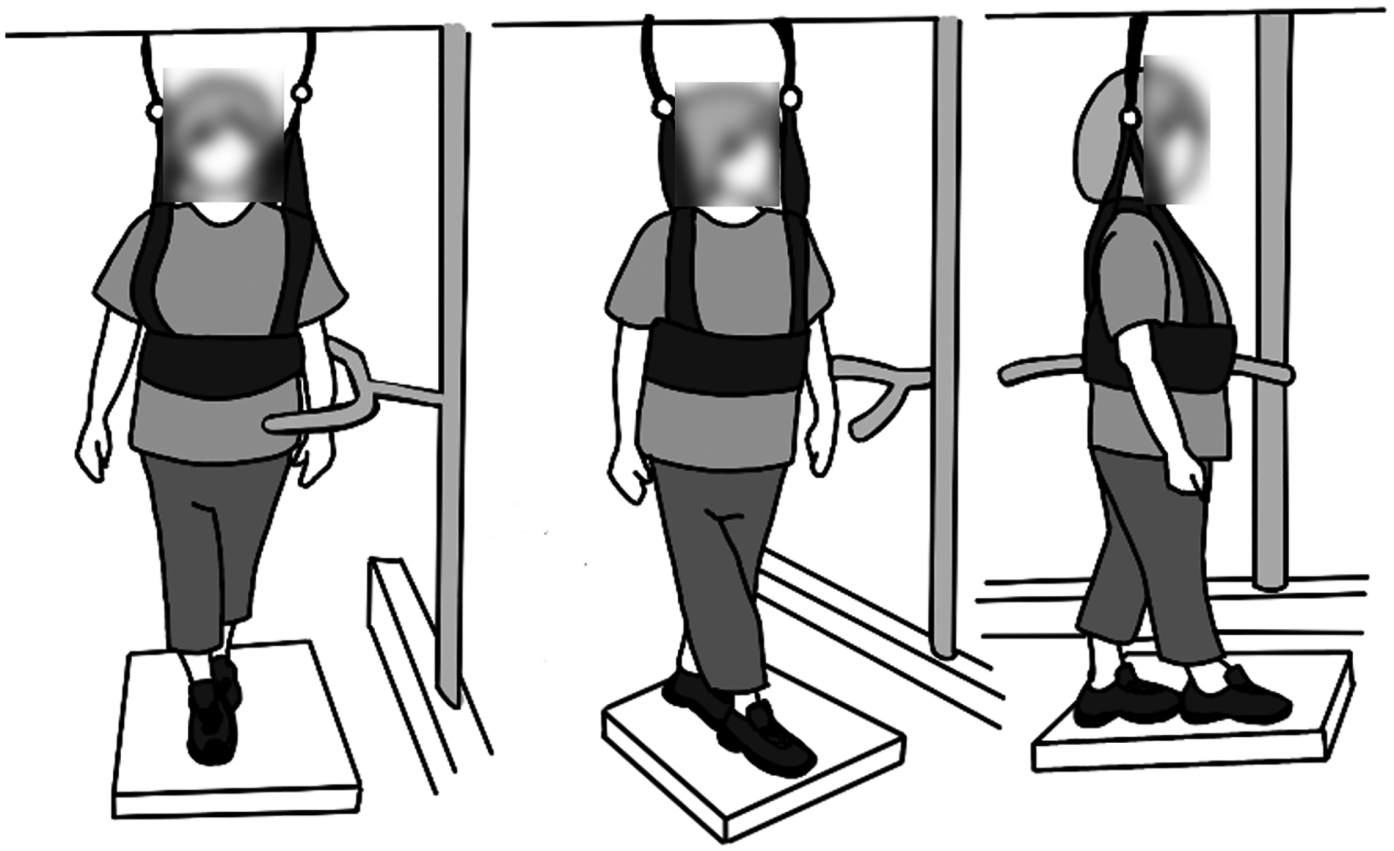
Layout of the video viewed by the PT participants. The video showed the coronal plane (left), sagittal plane (right), and the plane midway between the coronal and sagittal plane (center).

All trials were video recorded from three angles such that the coronal plane, the sagittal plane, and the plane midway between the coronal and sagittal plane were visible. PT participants viewed de-identified videos showing the balance participant from all three angles of each trial once (see Figure 1) and provided per-trial intensity ratings *via* an electronic PDF form. Additionally, after viewing the videos for all three trials, they rated the overall intensity for that specific exercise. A PT participant rated all 45 trials from each balance participant for whom they were a rater, and each balance participant's videos were rated by 3–5 randomly selected PT participants. Trial order was not randomized so that the assessments could more closely reflect clinical practice in which patients often repeat multiple repetitions of the same exercises one after the other.

Twenty one per-trial PT ratings were excluded due to missing data. Consequently, 450 per-trial and 150 per-exercise self-ratings and 1,935 per-trial and 645 per-exercise PT ratings were analyzed.

### 2.4. Data analysis

**Figure 2:**
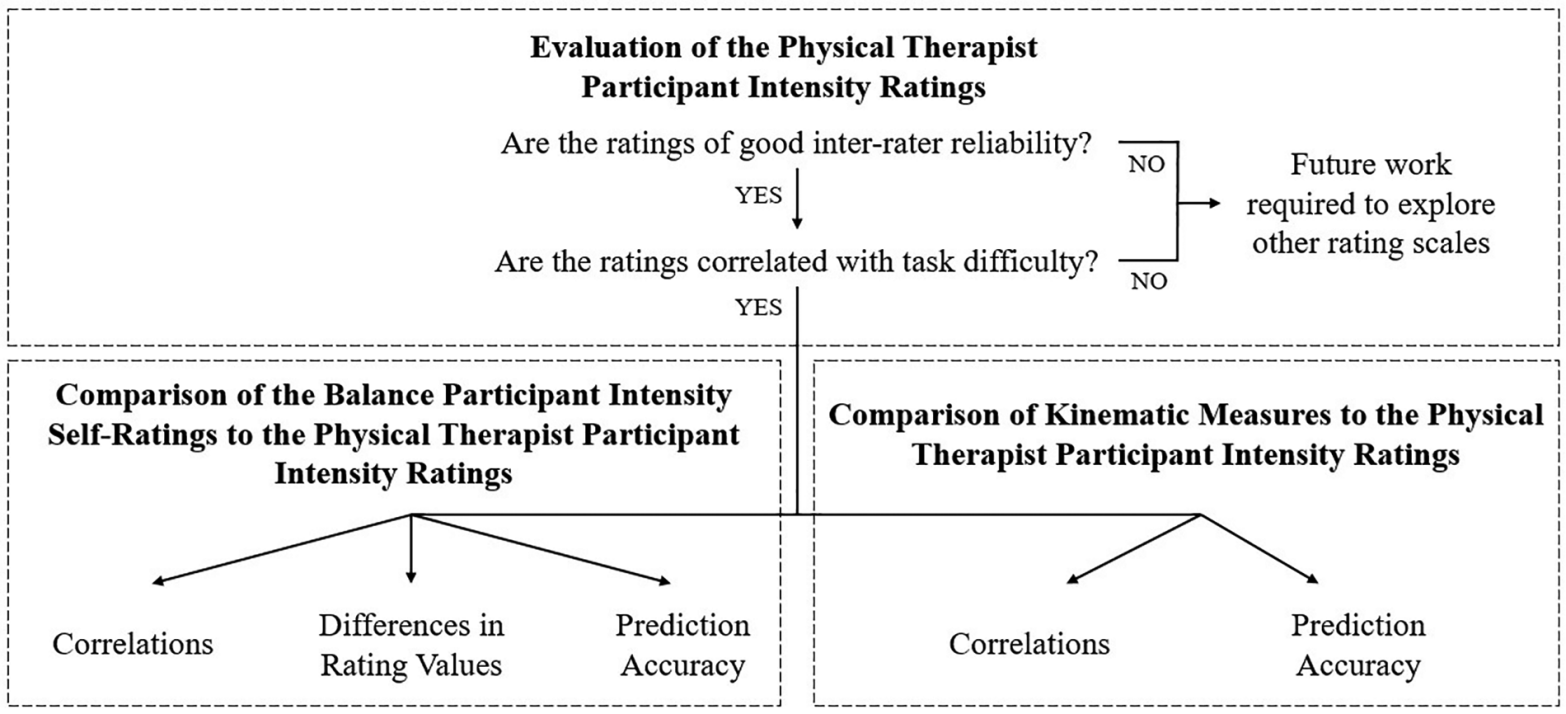
An overview of the analysis components. After assessing the PT ratings, these ratings were then compared to self-ratings or kinematic measures.

MATLAB (R2021b, Mathworks, Natick, MA) was used to process and analyze the data according to the plan summarized in Figure 2. The ordinal rating data were treated as continuous throughout without impact on the statistical results, as is supported by recent literature (67–71).

#### 2.4.1. Evaluation of physical therapist participant intensity ratings

Because to our knowledge only one prior study examined PT ratings and reported limited separation (62), we first examined inter-rater reliability and the relationship between ratings and task difficulty. High inter-rater reliability was a prerequisite for further analysis due to its association with scale validity, study replicability, and the accuracy of alternative assessments. Reliability is an indicator of a scale's internal structure and as such, is a prerequisite to the scale's validity (72, 73). Low inter-rater reliability may also obfuscate true variation and therefore negatively affect replicability (74–76). Low inter-rater reliability would also impose a ceiling on the accuracy of alternative assessment methods (e.g., self-assessments or kinematic measures) because the alternative assessment could at best agree with only the majority PT rating. Similarly, weak correlation between the PT intensity ratings and task difficulty would suggest that the ratings scale may not be capturing intensity and may accidentally be capturing a different construct.

The effect of increasing exercise difficulty on PT ratings was evaluated by regressing the ratings against four dimensions of exercise difficulty: surface (coded 1: firm, 2: foam), stance (coded 1: feet apart, 2: feet together, 3: partial heel to toe, 4: heel to toe, 5: single leg), visual input (coded 1: eyes open, 2: eyes closed), and head movements (coded 1: no movements, 2: pitch or yaw movements). These dimensions and their relative difficulties were based on information from Klatt et al. (57). The regression was performed using a linear mixed model in which intensity rating was the outcome, one dimension of exercise difficulty was a fixed effect, and the specific balance participant and PT participant were random effects (rating = intercept + dimension of exercise difficulty + balance participant + PT participant + error) (53).

Additionally, the inter-rater reliability of the per-trial PT ratings was evaluated as ICC(2,1) *via* a linear mixed effects model that decomposed the variance. Fixed effects included the intercept and trial number (i.e., 1, 2, or 3), and random effects included all possible interactions between trial number, exercise, balance participant, and PT participant. The intraclass correlation coefficient (ICC) of the PT per-trial ratings was then calculated by dividing the variance of the subset of random effects that included the rater by the variance from all sources (77, 78). The inter-rater reliability of the PT per-exercise ratings was calculated using the same approach without the inclusion of random effects associated with trial number (since no trial number was associated with the exercise as a whole). ICCs less than 0.5 indicated poor reliability, ICCs between 0.5 to 0.75 indicated moderate reliability, ICCs between 0.75 to 0.9 indicated good reliability, and ICCs greater than 0.9 indicated excellent reliability (78).

#### 2.4.2. Comparison of balance participant intensity self-ratings to physical therapist intensity ratings

The overall strength of the relationship between the per-exercise PT and self-ratings was quantified by the Spearman correlation coefficient. The systematic and random differences between the PT and self-ratings were then assessed by examining a contingency table of the two rating modes and quantified by the average and variance of the per-trial rating differences. The structure of these differences was further explored by using a paired t-test to compare the sample variances among the three per-trial PT ratings and the three per-trial self-ratings.

The overall effects of these differences were assessed by calculating how frequently a PT participant's rating could be accurately predicted given the self-rating, i.e., the accuracy when using self-ratings to predict per-trial and per-exercise PT ratings. The predictions were made using a linear model of the per-trial or per-exercise self-ratings.

#### 2.4.3. Comparison of kinematic measures to physical therapist intensity ratings

Correlations between trunk kinematics and PT ratings were assessed by first calculating kinematic features and then conducting correlation tests.

Prior to feature calculation, the IMU angular position data were filtered using a second order Butterworth filter with a 3 Hz cutoff frequency (53). Kinematic features were then calculated including: root mean square (RMS) of the angular position (Phi RMS, degrees; Phi_Angle^2^ = AP_Angle^2^ + ML_Angle^2^ = Pitch^2^ + Roll^2^), RMS in the anterior-posterior (AP) direction (AP RMS, degrees; i.e., Pitch), RMS in the medial-lateral (ML) direction (ML RMS, degrees; i.e., Roll), mean sway velocity (MV, degrees/s), path length as computed by the sum of the magnitude of the differences between roll and pitch sway data points (degrees), and area of a 95th percentile confidence interval elliptical fit to the roll and pitch sway data (i.e., elliptical area; EA; degrees^2^) (26, 79). Smaller RMS and EA values have been associated with better balance performance (80–83), while reports of the relationship between MV and balance performance are mixed (81, 83, 84). As suggested by Alsubaie et al. and Schieppati et al., the logarithm of each of these kinematic features were also considered (53, 85).

The strength of the relationship between each kinematic feature and the PT ratings was then evaluated using correlation as calculated using a linear model with rating as the outcome variable, kinematic feature as a fixed effect, and balance participant as a random effect (rating = intercept + feature + balance participant + error) (53). The marginal correlation was calculated as (86):

(1) $$R^{2}=\frac{\sigma_{f}^{2}}{\sigma_{f}^{2}+\sigma_{\alpha }^{2}+\sigma_{\varepsilon }^{2}}$$

(2) $$\sigma_{f}^{2}=var(\beta x)$$

Where $\sigma_{f}^{2}$ is the marginal variance calculated from the multiplication of the feature coefficient β and the feature x, $\sigma_{\alpha }^{2}$ is the variance of the balance participant random effect, and $\sigma_{\varepsilon }^{2}$ is the error variance.

The overall effects of these various correlations were assessed by calculating how frequently a PT participant's rating could be accurately predicted given the sway kinematics, i.e., the accuracy when per-trial or per-exercise PT ratings were predicted using a linear combination of all kinematic features from the trial or all three trials, respectively.

Finally, the overall strength of the relationship between sway kinematic measures and intensity ratings was assessed using the Spearman correlation between the kinematics-predicted and per-trial or per-exercise PT ratings.

## 3. Results

### 3.1. Evaluation of physical therapist participant intensity ratings

The per-trial and per-exercise PT ratings were both of good inter rater reliability (per-trial: ICC = 0.868, per-exercise: ICC = 0.860). Additionally, increases in exercise difficulty were significantly correlated with increases in per-trial and per-exercise PT ratings (see Table 2).

**Table 2:** Linear relationship between per-trial and per-exercise PT ratings and aspects of exercise difficulty. * denotes significance (*p* < 0.05).

Exercise Aspect	Per-Trial Intensity Ratings		Per-Exercise Intensity Ratings	
	Estimate [95% CI]	*p*-Value	Estimate [95% CI]	*p*-Value
Surface	0.366 [0.241, 0.492]	≤0.001*	0.354 [0.137, 0.571]	≤0.001*
Stance	0.607 [0.561, 0.653]	≤0.001*	0.606 [0.526, 0.686]	≤0.001*
Visual Input	0.584 [0.455, 0.713]	≤0.001*	0.631 [0.409, 0.853]	≤0.001*
Head Movements	−0.337 [−0.465, −0.210]	≤0.001*	−0.300 [−0.520, −0.081]	0.007*

### 3.2. Comparison of balance participant intensity self-ratings to physical therapist intensity ratings

The per-exercise self-ratings were significantly correlated with the PT ratings [r = 0.769, 95% CI = (0.735, 0.799), *p* ≤ 0.001].

**Figure 3:**
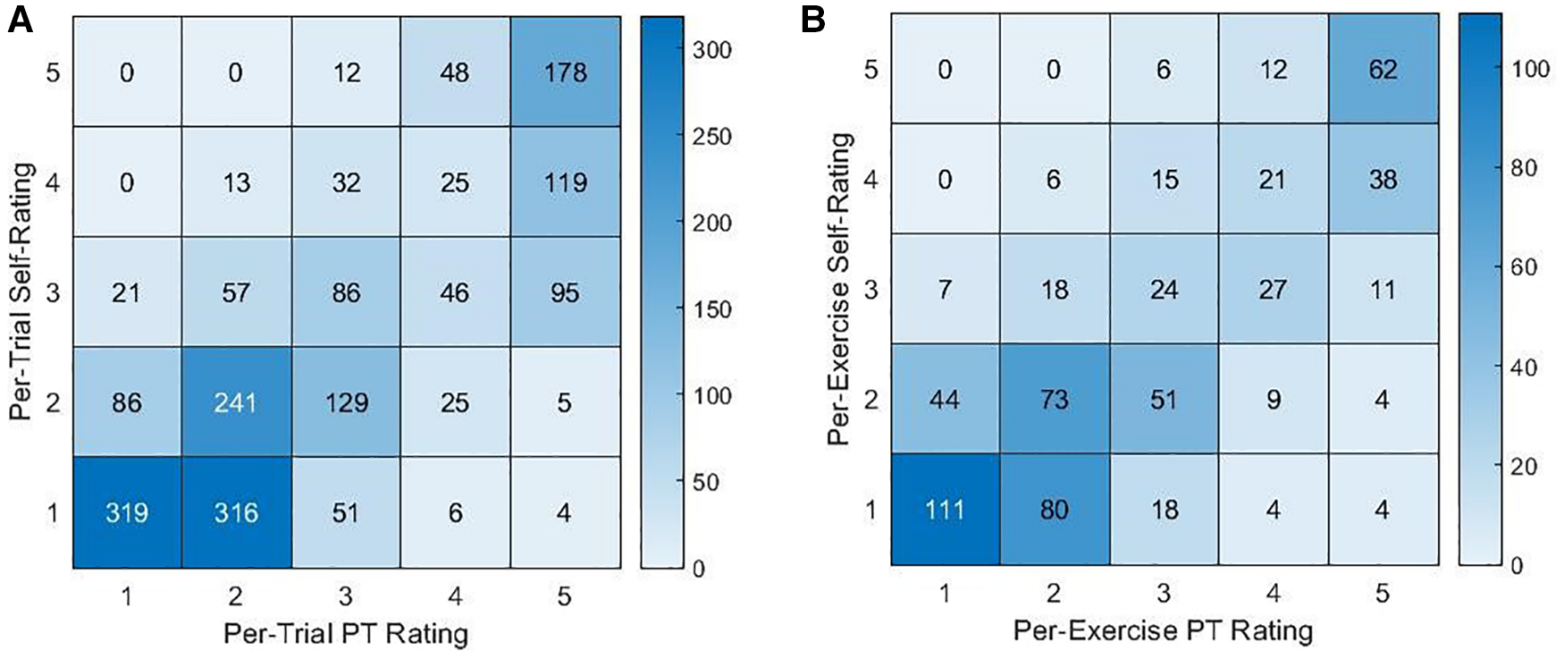
Contingency tables for the counts of PT ratings and (**A**) per-trial or (**B**) per-exercise self-ratings.

As shown in Figure 3, self-ratings agreed with PT ratings most often for ratings of a 5 (per-trial and per-exercise self-ratings of a 5 agreed with PT ratings 74.8% and 77.5% of the time, respectively). Self-ratings of a 4 also most often corresponded to a PT rating of a 5 (per-trial: 63.0%, per-exercise: 47.5% for self-ratings of a 4) while self-ratings of a 3 corresponded to various PT ratings, and self-ratings of a 2 or 1 most often corresponded to PT ratings between 1 and 3.

However, the per-trial and per-exercise self-ratings were lower than the PT ratings [absolute per-trial rating difference: 0.385, per-exercise: 0.314, 95% CI = (0.048, 0.581), *p* = 0.021]. Additionally, the standard deviations of the difference in per-trial or per-exercise ratings were 0.920 and 0.961 [0.910, 1.015], respectively. Self-ratings exhibited a smaller variance between trials of the same exercise than the PT ratings did [0.102, 95% CI = (0.049, 0.154), *p* < 0.001].

As a result, when using self-ratings to linearly predict per-trial or per-exercise PT ratings, the accuracies were 46.6% and 45.9%, respectively.

### 3.3. Comparison of kinematic measures to physical therapist intensity ratings

**Figure 4:**
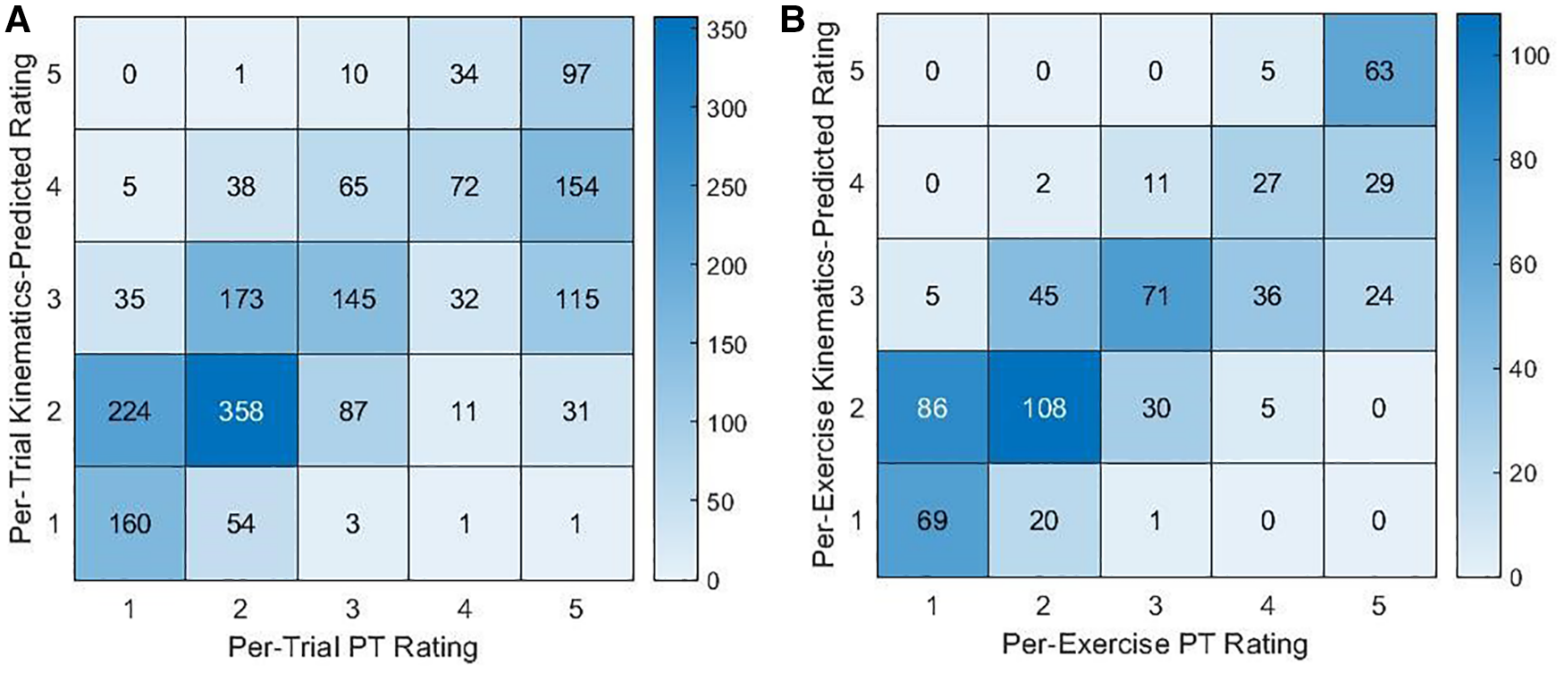
Contingency tables for the counts of PT ratings and (**A**) per-trial or (**B**) per-exercise kinematics-predicted ratings.

The kinematic features were significantly correlated with PT ratings (see Table 3). When using all kinematic features together to predict PT ratings, the resulting predictions were significantly correlated with per-trial or per-exercise PT ratings (per-trial: r = 0.698, 95% CI = [0.675, 0.721], *p* ≤ 0.001; per-exercise: r = 0.826, 95% CI = [0.799, 0.850], *p* ≤ 0.001) and agreed with the PT ratings 43.0% and 52.4% of the time, respectively. As shown in Figure 4, kinematics-predicted per-exercise ratings agreed with PT ratings most often for ratings of a 5 (kinematics-predicted ratings of a 5 agreed with PT ratings 92.7% of the time). Kinematics-predicted per-exercise ratings of a 4 also most often corresponded to a PT rating of a 5 (42.0% for kinematics-predicted ratings of a 4) but also often corresponded to a 4 (39.1% for kinematics-predicted ratings of a 4). Kinematics-predicted ratings of a 3 corresponded to various PT ratings, and self-ratings of a 2 or 1 most often corresponded to PT ratings of a 2 or 1.

**Table 3:** Correlation coefficients between kinematic features and per-trial PT ratings. RMS denotes root mean square, and MV denotes mean velocity. * denotes significance (*p* < 0.05).

Kinematic Feature	No Transform		Log Transform	
	R	*p*-Value	R	*p*-Value
Phi RMS	0.588	≤0.001*	0.724	≤0.001*
AP RMS	0.482	≤0.001*	0.622	≤0.001*
ML RMS	0.657	≤0.001*	0.735	≤0.001*
Phi MV	0.609	≤0.001*	0.728	≤0.001*
AP MV	0.548	≤0.001*	0.664	≤0.001*
ML MV	0.618	≤0.001*	0.739	≤0.001*
Path Length	0.651	≤0.001*	0.731	≤0.001*
Elliptical Area	0.349	≤0.001*	0.736	≤0.001*

## 4. Discussion

### 4.1. Evaluation of physical therapist participant intensity ratings

The per-trial and per-exercise PT ratings were of good inter-rater reliability, supporting that the study results may be replicable and that it would be possible for the alternative assessments to accurately predict PT ratings. Higher ratings were also associated with higher exercise difficulty along three out of four dimensions (57) when accounting for differing individual balance participant, suggesting that the ratings do reflect task intensity. While higher ratings were not associated with more difficult head movement conditions, this could be due to the majority of balance participants having no vestibular dysfunction and consequently being less affected by head movement. Similarly, Alsubaie et al. and Anson et al. reported significant associations between balance exercise difficulty and self-ratings of exercise intensity or postural stability (5, 53).

However, it is important to note that while the descriptors for PT ratings 1, 2, and 3 referenced amount of sway, prior research has reported variable sway results for people with balance impairments (e.g., sometimes increased sway, sometimes decreased sway) (87–90). Increased sway may be interpreted as poorer control over the body's position while decreased sway may reflect an adaptive strategy in which people stiffen in order to simplify the balance task by reducing the degrees of freedom of movement (91). As a result, future work may investigate alternative scale descriptors.

### 4.2. Comparison of balance participant intensity self-ratings to physical therapist intensity ratings

While the self-ratings were strongly correlated with the PT ratings, the self-ratings were an imperfect approximation of PT ratings. As has been described by the Dunning-Kruger effect and reported in other contexts (92–94), balance participants overestimated the quality of their performance, self-rating the exercise as less intense than the ratings provided by the PT participants. Accounting for this offset may therefore improve the agreement between PT and self-ratings. However, the significant standard deviation in the rating difference suggests that a simple adjustment is inadequate when using self-ratings as a support to PT assessment. The standard deviation in rating difference may partially be driven by the lower variation in self-ratings between the three trials of an exercise compared to the PT's ratings. This may reflect differences in rating strategy such as the balance participants more heavily considering their performance of prior trials when rating the current trial. The variation in rating difference may also be due to differences in participants' determinations of what constituted a loss of balance, imperfect observations of the performance (e.g., a PT participant not seeing or a balancer participant not noticing movement of the feet), imperfect recollection of the performance when providing the rating, or accidental misuse of the scale (e.g., selection of a 1 when intending to select a 5). These factors likely played some role as is reflected in imperfect agreement for ratings of 4 or 5 and occasional confusion between ratings of 1 and 5 (see Figure 3).

As a result of these differences, predicted PT ratings based on self-ratings exhibited moderately low accuracy. The common confusion between ratings of a 4 or a 5 as well as between ratings of a 1 or a 2 and the overall ambiguity for ratings of a 3 might suggest that balance participants are better able to distinguish between two levels of balance intensity (e.g., higher-lower). The confusion between ratings of 1, 2, or 3 may also reflect the aforementioned scale limitation in which the PT's descriptors reference sway, which may indicate either good or poor balance. As a result of these issues, if using self-ratings to support intensity assessment during telehealth or other contexts in which traditional PT assessment might be difficult, self-ratings might be used to suggest higher or lower intensity but may not suggest more granular levels of intensity.

### 4.3. Comparison of kinematic measures to physical therapist intensity ratings

The kinematics-predicted intensity ratings were also imperfect yet informative approximations of PT ratings, as reflected by strong correlations, but moderately low prediction accuracies. The correlations between kinematic features and per-trial PT ratings were higher in the ML direction than in either AP or Phi directions, suggesting that, on average, PT participants based their evaluation of balance intensity more heavily upon movement in the ML direction. Similar correlation values as well as higher correlations in the ML direction were also reported by Alsubaie et al. in two investigations of intensity self-ratings (R between 0.56 and 0.88) (53, 56). The correlation coefficients were slightly higher when using the logarithm of the kinematic feature compared to the untransformed values, especially so for EA. Higher correlation between ratings and the logarithm of a kinematic feature than with the original measure were also reported by Alsubaie et al. regarding intensity self-ratings and by Scieppati et al. and Anson et al. regarding postural stability self-ratings (5, 53, 85).

Agreement between kinematics-predicted intensity ratings and PT ratings was higher than for self-ratings, resulting in especially high rates of agreement for kinematics-predicted ratings of a 5. If using kinematics to support PT assessments during telehealth or other applications, kinematics may be more heavily considered during high-intensity exercises with ratings near a 5 or for low-intensity exercises with ratings of a 1 or 2.

In this first and necessary study on the differences between alternative modes of balance intensity assessment, both self-ratings and sway kinematics were shown to have the potential to support PT assessment of balance exercise intensity when expert visual assessment is difficult or limited, with sway kinematics being more accurate than self-ratings. Intensity assessments might be used in conjunction with frameworks for exercise progression such as the one proposed by Klatt et al. so that balance training can be remotely assessed and progressed over time (57). However, the results from this study suggest that self-ratings and conventional trunk kinematic measures cannot fully replicate or substitute for PT assessments. Based on our findings, PTs might employ self-ratings and/or sway kinematic measures to supplement their partial visual assessments during telerehabilitation or home-based training. If doing so, our preliminary results suggest that the PT should consider self-ratings as indications of high versus low intensity rather than five levels, and kinematic measurements should be more heavily considered during exercises at intensity extremes (e.g., very high or very low). Combining self-ratings of intensity with kinematic measures as well as employing more complex techniques may also lead to more accurate predictions, as reported by Bao et al. and Kamran et al. (50, 51). Further exploration of related machine learning architectures and expanded datasets might further improve the prediction accuracy.

Limitations of this study included the performance of a subset of balance exercises (i.e., static standing) and a subset of balance participants that would benefit from balance training (i.e., older adults and adults with vestibular disorders). Future work could include the exploration of alternate scale descriptors without reference to amount of sway, evaluation of additional balance-related pathologies and balance exercises, evaluation of the effects of PT experience level, analysis of full-body kinematics, and the use of data science methodologies such as machine learning approaches to capture additional trends in the kinematic data.

## 5. Summary

This study examined the relationship between PT intensity ratings, self-assessments, and kinematic measures. Both self-ratings and trunk kinematic features correlated significantly with PT ratings of intensity. Based on the findings from this study, self-ratings may better distinguish between two levels of intensity (i.e., higher-lower) than five levels of intensity. Furthermore, kinematics-predicted intensity ratings at either extreme (i.e., 1: steady or 5: loss of balance) may be more reliable indicators of intensity than the other ratings in the evaluated scale. These findings suggest that self-assessments and kinematic measurements may support PT intensity assessments during contexts in which visual assessment is difficult (e.g., during telerehabilitation).

## Data Availability

The raw data supporting the conclusions of this article will be made available by the authors, without undue reservation.
